# Reply to Narayan P

**DOI:** 10.1093/icvts/ivaf201

**Published:** 2025-09-04

**Authors:** Andreas Schaefer

**Affiliations:** Department of Cardiovascular Surgery, University Heart and Vascular Center Hamburg, University Medical Center Hamburg-Eppendorf, 20249 Hamburg, Germany

We read with great interest the letter-to-the-editor of Pradeep Narayan^1^ regarding our work “Coronary artery bypass grafting using both internal mammary arteries-a safe concept for surgical training”.^2^ Herein, the author comments on methodological concerns regarding our work. While we agree with the fact that the raised points are valid, we want to emphasize that all these points are proactively addressed throughout the work, and specific analyses were performed to further clarify these issues, in particular:

1. In the supplementary appendix, the total CABG cases per surgeon and annual procedure volume per surgeon are documented; furthermore, a CUSUM analysis was performed to address this exact point (outcome per case load). In addition, we want to emphasize that all residents of the authors’ center perform CABG using BIMA from the start of surgical training as documented in the manuscript (“At the authors’ institution, all residents perform CABG using two arterial grafts with an aortic no-touch technique from day 1 of surgical training when anatomically and patient specific suitable.”)^2^
2. The mentioned selection bias is of particular importance to understand the documented results and was also addressed several times throughout the manuscript (“It is important to highlight that staff surgeons were more frequently involved in performing technically more challenging procedures like complex bypass anastomoses in terms of higher rates of sequential grafting techniques compared to residents.”; “Due to the retrospective nature of the herein conducted study, limitations are inherent in the study design: patients were not randomized to a specific treatment; therefore, patient preselection with hidden confounders may apply. Due to absence of objective selection criteria, there may be a possible bias of the selection process in terms of absence of objective selection patterns.”).^2^ Furthermore, we emphasize several times that CABG using BIMA performed by residents should be done under supervision and that mainly lower-risk patients requiring fewer anastomoses were operated by residents.
3. A limitation of our study is the limited statistical power to detect small differences in rare adverse events such as mortality or stroke, particularly given the relatively low number of events in both groups. However, the absolute event rates were very low and similar between surgeries performed by residents and staff surgeons. Moreover, the observed effect sizes were negligible, suggesting that any potential differences—if they exist—are unlikely to be of clinical significance. While these results should be interpreted cautiously, the findings support the notion that, in a supervised setting, surgeries performed by residents can achieve comparable postoperative outcomes to those performed by experienced staff surgeons, even if the study was not powered to detect very small differences.

In conclusion, as mentioned in the original manuscript, the goal of the presented work was to show that CABG using BIMA is safely performable by residents when done under supervision and with prior adequate patient selection, which is underlined by the very low event rates in both investigated patient cohorts.
